# An Overview of CD133 as a Functional Unit of Prognosis and Treatment Resistance in Glioblastoma

**DOI:** 10.3390/curroncol30090601

**Published:** 2023-09-07

**Authors:** Thomas Joyce, Sarisha Jagasia, Erdal Tasci, Kevin Camphausen, Andra Valentina Krauze

**Affiliations:** Radiation Oncology Branch, Center for Cancer Research, National Cancer Institute, NIH, 9000 Rockville Pike, Building 10, CRC, Bethesda, MD 20892, USA; thomas.joyce@nih.gov (T.J.); sarisha.jagasia@nih.gov (S.J.); erdal.tasci@nih.gov (E.T.); camphauk@mail.nih.gov (K.C.)

**Keywords:** CD133, stem cell marker, glioblastoma, chemo/radioresistance, recurrence, omics

## Abstract

Biomarkers for resistance in Glioblastoma multiforme (GBM) are lacking, and progress in the clinic has been slow to arrive. CD133 (prominin-1) is a membrane-bound glycoprotein on the surface of cancer stem cells (CSCs) that has been associated with poor prognosis, therapy resistance, and tumor recurrence in GBM. Due to its connection to CSCs, to which tumor resistance and recurrence have been partially attributed in GBM, there is a growing field of research revolving around the potential role of CD133 in each of these processes. However, despite encouraging results in vitro and in vivo, the biological interplay of CD133 with these components is still unclear, causing a lack of clinical application. In parallel, omic data from biospecimens that include CD133 are beginning to emerge, increasing the importance of understanding CD133 for the effective use of these highly dimensional data sets. Given the significant mechanistic overlap, prioritization of the most robust findings is necessary to optimize the transition of CD133 to clinical applications using patient-derived biospecimens. As a result, this review aims to compile and analyze the current research regarding CD133 as a functional unit in GBM, exploring its connections to prognosis, the tumor microenvironment, tumor resistance, and tumor recurrence.

## 1. Introduction

Glioma is the most common type of primary central nervous system tumor and is characterized by a poor prognosis. Glioblastoma multiforme (GBM) is the most aggressive type of glioma and is defined by its short survival [1]. Despite the standard of care consisting of surgical removal followed by chemoradiation therapy, the median survival length of GBM has been found to be only 14.6 months, with a two-year survival rate of 26.5% [2]. When only using radiotherapy, however, these values have been found to decrease to 12.1 months and 10.4%, respectively [2]. One of the primary reasons for this poor prognosis is the ability of GBM to infiltrate throughout the brain making complete surgical removal nearly impossible and allowing for resistance and recurrence [3].

Increasing evidence has attributed the resistance and recurrence capabilities of GBM to a small population of cells exhibiting stem-like characteristics known as cancer stem cells (CSCs). CSCs are undifferentiated cells that can divide and form two daughter cells: one that retains stem-cell characteristics and one that experiences differentiation, therefore propagating tumor populations [4]. Compared to other cancer cells, CSCs can increase tumorigenic potential due to their unlimited proliferation, self-renewal abilities, and resistance tactics, all of which continue to be explored. These traits result in chemo- and radioresistance, allowing for tumor regrowth following conventional therapies [5]. As a result, research has focused on identifying CSCs using markers in an attempt to uncover the potential mechanisms behind these processes. CD133 has been one of the most commonly identified and utilized CSC markers, and its existence has been found in various other types of cancer as well, including breast [4], lung [6], stomach [7], prostate [8], and ovarian [9], amongst others.

**Figure 1:**
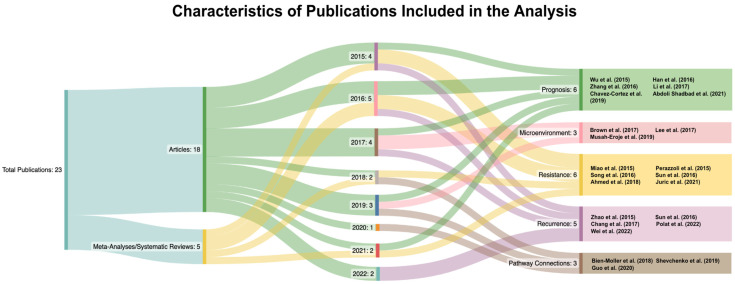
Modified Sankey diagram sorting the publications used into publication type, year, and subgroup [9,10,11,12,13,14,15,16,17,18,19,20,21,22,23,24,25,26,27,28,29,30,31].

CD133, also known as prominin-1, is a membrane-bound glycoprotein on the surface of stem cells. With regard to GBM, it has been affiliated with poor prognosis, therapy resistance, and tumor recurrence [4]. However, the majority of recent research has focused mainly on using CD133 to identify CSCs, rather than on how CD133 potentially operates and what pathways it may be involved in to target for optimal treatment approaches. Due to its largely unmatched presence on CSCs and its affiliation with these significant tumor characteristics, however, CD133 in particular has earned enough credibility for its contributions to be explored. As a result, there is an emerging field of research that investigates CD133 as a functional unit, which is the focus of this review. This review only includes research published after 2014 focused on CD133 itself, rather than its use simply as a biomarker, to provide a presentation of the current field of research and how its conclusions could lead to clinical applications. Table 1 provides a brief synopsis of the significant publications utilized in this review, and through analysis of these articles, Figure 1 displays the various subcategories that they were sorted into. This review aims to present a comprehensive overview of CD133 and its role in glioma, including its possible prognostic correlations, microenvironmental relations, involvement in therapy resistance and tumor recurrence, and pathway connections.

## 2. CD133 and GBM Prognosis

Despite CD133 being used consistently to identify the presence of CSCs, there is growing research involving its relationship to prognosis and survival rates. Several recent analyses have investigated the correlation between high CD133 expression and overall survival (OS) and progression-free survival (PFS). When conducting these analyses, both Wu et al. (32 studies totaling 1490 patients) and Zhang et al. (10 studies totaling 715 patients) observed an association between high CD133 expression and decreased OS as well as decreased PFS [9,11]. Wu et al. also used meta-analyses to specify this correlation, discovering that these results were specific to WHO IV gliomas, but not WHO II-III [9]. Han et al. reported similar findings when analyzing the outcomes of 1535 patients across 21 studies, concluding that high CD133 expression was affiliated with poor 2-year OS and PFS. This meta-analysis also found that these findings were specific only to high-grade WHO gliomas, specifically III and IV [10]. A final meta-analysis, by Abdoli Shadbad et al., also confirmed these findings after investigating 1086 patients across 12 studies and discovering that overexpression of CD133 was correlated with inferior PFS for patients with high-grade gliomas [14]. These findings indicate that CD133 expression levels may be correlated with the severity of the diagnosis, which may mean that it can provide prognostic information. However, it is significant to note that there was immense overlap between studies in these meta-analyses, which may have influenced why they produced similar results.

As all of this research has linked the high expression of CD133 with poor prognosis [9,10,11,14], Li et al. expanded upon this, discovering a high expression of CD133 particularly when there is a low expression of HOX genes [12]. This implied a possible interdependent link between CD133 and HOX genes, suggesting that overexpression of only one of these may be sufficient to perpetuate glioma progression. However, the certification of each of them independently being related to patient survival outcomes also indicated the possibility for combined analyses when exploring glioma prognosis using CD133 [12].

It is difficult, however, to attribute these prognoses specifically to CD133 as poor prognosis is incredibly common in GBM. As a result, investigations specifically targeting CD133 can provide insight into its true relationship to prognosis. Studies aimed at this are beginning to emerge, such as a recent study by Chavez-Cortez et al. in which an immunotoxin for CD133 was created from IgY antibodies from birds. Upon applying this immunotoxin both in vitro and in vivo, it was determined that the viability of glioma CSCs was reduced by 55% [13]. When this was done intratumorally, however, a 50% reduction in tumor size was also determined. As a result, the targeting of CD133 in particular caused reductions in both tumor size and progression showing that it may be an active factor in OS and PFS [13]. Although many more studies are needed in this space, these findings imply a potential direct connection between prognosis and the expression of CD133 cells.

## 3. CD133 and the Tumor Microenvironment

**Figure 2:**
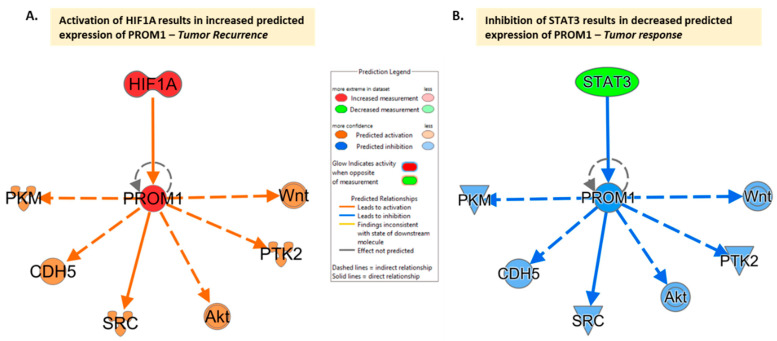
CD133 (PROM1) interaction network limiting interaction to molecules in cancer based on the IPA molecule activity prediction ((QIAGEN Inc., https://www.qiagenbioinformatics.com/products/ingenuitypathway-analysis) accessed on 24 July 2023) [32]. Network comparison analysis with (**A**) Activation of HIF1A (e.g., under hypoxia conditions) results in increased predicted expression of PROM1 and the hypothesis is that this drives tumor recurrence. (**B**) Inhibition of STAT3 results in decreased predicted expression of PROM1 and the hypothesis is that this can lead to tumor response. The same downstream mediators appear to be involved, given current evidence, with PROM1 activation or inhibition driving activation/inhibition of downstream molecules. The extended interaction network is presented in Figure A2.

Recent research has explored the impact that microenvironmental factors have on CD133, suggesting the possibility that changes in the microenvironment may mediate its expression. The exploration of the impact of microenvironmental factors has focused mainly on hypoxia, as rapid tumor proliferation leads to diminished blood supply causing hypoxia and necrosis, as well as driving the stem cell state. As a result, investigating how cells survive in this environment, while still allowing GBM in particular to maintain resistance to conventional therapies, is crucial for potential treatment approaches. Musah-Eroje and Watson explored this correlation by placing three GBM cell lines in 1% oxygen microenvironments and discovered that there was a significant upregulation of CD133 under hypoxic conditions [17] (Figure 2A). Similar research has confirmed this increased expression due to hypoxia in the microenvironment [15,16,21], with Ahmed et al. confirming this in both 2D and 3D models [22].

However, these discoveries are not solely focused on the impact of hypoxia, but instead on the possible inferences that can be made utilizing these observed effects. Musah-Eroje and Watson also discovered the reversibility of CD133 expression, suggesting that there may be a more significant linkage between CD133 expression and the microenvironment [17]. Their findings imply that gene expression is based on the microenvironment and that GBM cells are adaptive to this environment, changing expression qualities in a dynamic manner. Therefore, this may provide insight into the movement of GBM cells as well as treatment mechanisms revolving around the genetic characteristics that control these changes in expression [17]. Brown et al. observed a similar change in phenotype based on microenvironmental conditions, witnessing the ability of CD133+ cells to transform into CD44+ cells. This ability was possibly linked to CD133+ cells actively cycling, allowing for this manipulation of phenotype [15].

In contrast, Lee et al. suggested that CD133 expression has the ability to modify the microenvironment, rather than the reverse. This study explored the relationship between CD133 and the IL-1β signaling pathway and the downstream chemokines associated with it [16]. Although this study did not present findings that this activation led to increased survival or proliferation, they did find an influx of neutrophils into the tumor microenvironment. Therefore, Lee et al. concluded that CD133-expressing glioma cells may be able to modify the tumor microenvironment by recruiting neutrophils and inflammatory cells to produce GBM resistance [16]. As a result, the relationship between CD133 expression and the microenvironment has been established, but the directionality of this relationship is still in contention.

## 4. CD133 and Resistance

The majority of the current research field has explored the correlation between CD133+ glioma cells and chemo- and radioresistance, however, there is a growing field that is investigating the contributions of CD133 as a functional unit in this resistance. Because of this, there is a currently increasing consensus regarding the involvement of CD133 in tumor resistance, but the mechanism by which this occurs is unclear, with various possibilities.

In regard to chemotherapy, there is still debate as to whether CD133 is involved in the resistance of glioma cells to temozolomide (TMZ), a common oral chemotherapeutic [19]. Although some research has discovered higher TMZ resistance with the presence of CD133 [22], the majority of more recent studies have not identified indications of its role in TMZ resistance when downregulating CD133 [19,21,23]. Despite this, Miao et al. investigated the role of CD133 in apoptosis specifically when treated with TMZ. It was discovered that following TMZ treatment, the apoptotic rate of CD133+/-U251R cells was much lower [18]. Along with this, the expression of the p53 upregulated modulator of apoptosis (PUMA), a crucial product of antitumor drugs that promotes apoptosis, was especially low in CD133+U251R cells, suggesting the possible downregulation of PUMA by CD133 expressing cells. As a result, the findings showed that the reintroduction of PUMA led to increased apoptotic rates, implying that the dual usage of PUMA and TMZ may be effective as a treatment approach [18].

Shifting to cisplatin, another chemotherapeutic, Ahmed et al. recently reported a possible direct involvement of CD133 in the resistance of GBM by also looking at apoptosis. The downregulation of CD133 resulted in an increase in cisplatin effectiveness ranging from two-fold, all the way to seven-fold in U251 cells [22]. This was attributed to the connection between CD133 and the phosphoinositide 3-kinase (PI3K) 85 kDa regulatory subunit, which initiates the PI3K/protein kinase B (Akt) pathway. This Akt pathway is an anti-apoptotic pathway, suggesting the direct involvement of CD133 in this resistance as a crucial and functional unit [22]. Also, cisplatin resistance of HIF mechanisms was displayed which also seemed to be dependent on CD133, indicating yet another way in which it may be involved in resistance [22]. To showcase the potential effects of the increased expression of CD133 (PROM1), its downstream effects were illustrated in IPA and it was observed that its increased expression predicted the coinciding increased expression of several of these downstream mediators [32] (Figure 2A, extended networks displayed in Figure A2).

Juric et al. also explored the effects of CD133 on apoptosis by investigating the connection between CD133 and cyclin-dependent kinases (CDKs). By using CDK inhibitors, it was found that sphere size was decreased and apoptosis increased when incorporated. As a result, this led to the potential explanation that CDK reduction would mean the presence of less anti-apoptotic agents, namely Mcl-1. This reveals yet another pathway that could be targeted when assessing effective new treatment techniques [23].

Rather than preventing apoptosis, Song et al. instead investigated the possible involvement of CD133 in the repair functions of GBM cells. This analysis confirmed that CD133+CSCs were associated with drug resistance, but a further inquiry into this mechanism revealed the communicative relationship between CD133 and Sox2, a neural stem cell marker [20]. It was determined that the removal of Sox2 decreased tumor cell population survival and sphere formation, seemingly indicating its involvement in drug resistance [20]. Due to the discovery of this interconnection, this is yet another avenue that can be explored.

Sun et al. focused more specifically on the role of CD133 in autophagy brought upon by nutrient-sparse microenvironments. Upon the discovery that cells expressing CD133 had an increased ability to survive, a connection between CD133 and mTOR was determined in which the two appeared to have a coinciding influence on autophagy [21]. From these findings and others in this research field, it was inferred that CD133 plays a potential role in creating phagophores in its fusion process as well as through the presence of CD133-containing particles in its composition. There also seems to be a potential role of CD133 in lysosome degradation processes [21]. Combined, both of these help to explain how CD133 contributes to the resistance of GBM, allowing for survival in difficult environments.

Although these all appear to be very different approaches to the involvement of CD133 in the resistance of GBM, what they display is the growing field focused on CD133 itself. This research presents the trend in the data to explore the mechanisms by which CD133 is involved in resistance and, by elucidating these, provide potential treatment approaches. More research is needed in this realm to provide more clarity as to which of these avenues is most promising, however, through this presentation, it is possible to see that apoptosis prevention, repair, and autophagy involvement are all emerging areas of focus.

## 5. CD133 and Recurrence

As with the research field focused on resistance, recurrence has become another growing area of interest due to the affiliation of recurrence with CD133+ cells. Across studies, it has been identified that recurrent GBM has higher expressions of CD133 when compared to the initial tumors [27]. This is significant because it shows that there are more CSCs in the recurrent tumor, possibly due to the contribution of CD133. Additionally, a recent meta-analysis found that the time to distant recurrence was decreased by the overexpression of CD133, implying its contribution [14]. However, the role of CD133 in this recurrence has only recently begun to be explored.

Defining the importance of CD133 with respect to recurrence was first necessary, and Chang et al. explored the exact tumorigenic changes that occurred when CD133 was present and removed. It was determined that when present, CSCs were able to form neurospheres, and these neurospheres contained markers for neural, embryonic, and pluripotent stem cells [26]. Additionally, when placed in nude mice, only the CD133+ population was able to grow tumors in vivo, as the CD133- population could not. It was also discovered that STAT3 was present at a higher rate in CD133+ cells and that when inhibited, the ability of these cells to form neurospheres was decreased. This showed that the inhibition of STAT3 may be a promising route for reducing the effectiveness of CD133 in tumorigenesis [26]. In fact, to showcase the potential effect of the decreased expression of STAT3 (Figure 2B, extended networks displayed in Figure A2) the immediate interactions of CD133 were illustrated in IPA and a decreased expression of PROM1 was observed which also acted to inhibit several downstream mediators [32].

**Figure 3:**
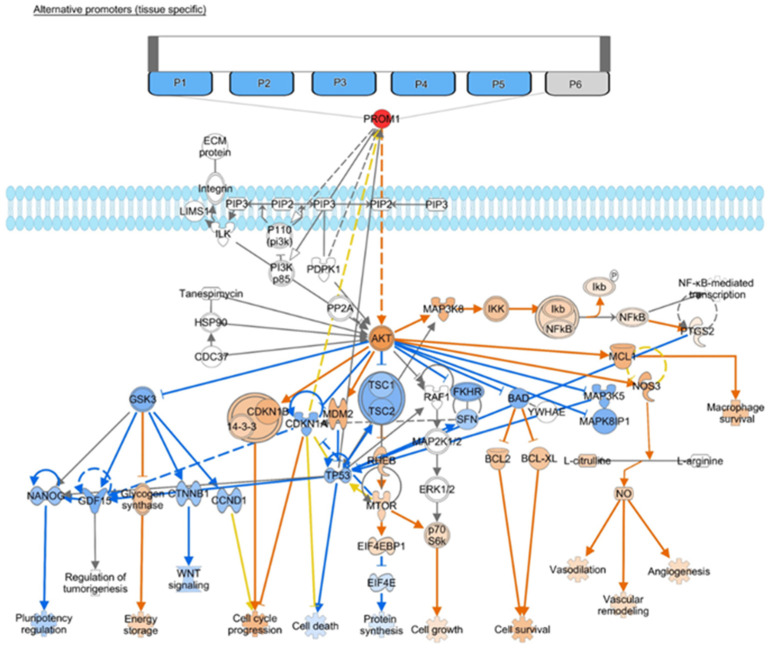
Alternative promoters (tissue specific) and the downstream effects of CD133 activation on the Akt pathway with implications for pluripotency, tumorigenesis, cell cycle effects, apoptosis and cell survival, protein synthesis, and angiogenesis based on IPA molecule activity prediction ((QIAGEN Inc., https://www.qiagenbioinformatics.com/products/ingenuitypathway-analysis) accessed on 24 July 2023) [32].

Sun et al. conducted one of the few studies that have aimed to expose the possible mechanistic involvement of CD133 in recurrence. The focus of this study was on the promoter regions of CD133, of which there are five shown in Figure 3, accompanied by the downstream effects of CD133 on the Akt pathways. Only three promoters (P1–P3) are on the CpG island, however, where they are exposed to methylation. It was determined that the hypomethylation of P2 specifically was required for CD133 transcription [25]. Additionally, when a demethylating agent was introduced, this increased the presence of CD133 mRNA. As a result, if this progression is to be proven, and the functional operations of CD133 solidified, this is a crucial insight into controlling its presence [25]. A study by Zhao et al. took a similar approach, finding that the insertion of the HMGB1 gene in CD133-expressing cells limited the multiplication of these cells while also inducing apoptosis [24]. As a result, the inhibition of this gene may be a component of how CD133 contributes to tumorigenesis.

Wei et al. also looked comprehensively at the mechanism that may be causing recurrence. It was determined that the *N*-glycan increased the interaction between CD133 and DNMT1, and research has suggested that the progression moves from CD133 to DNMT1 [28]. This interaction then increased p21 and p27, which contributed to CSC quiescence: when cells are nonproliferating and frozen in the G0 state for survival. Further research is needed, however, to determine how this interaction moves CSCs from the quiescence to the proliferating state. From this, however, Wei et al. was able to conclude that CD133 has an essential role in quiescence, recurrence, and tumorigenesis. Along with this, the inhibition of the CD133–DNMT1 interaction made CSCs more vulnerable to TMZ treatment [28].

Similar to resistance, how CD133 specifically contributes to recurrence is still debated as the current research field seeks to propose and confirm its mechanistic involvements. However, it has been made increasingly clear that its presence is central to the reformation of GBM tumors through neurosphere formation. These various pathway connections also imply that CD133 plays a significant role in this process, and that, once elucidated, could be an integral target when creating treatment approaches.

## 6. CD133-Relevant Linkages

**Figure A1:**
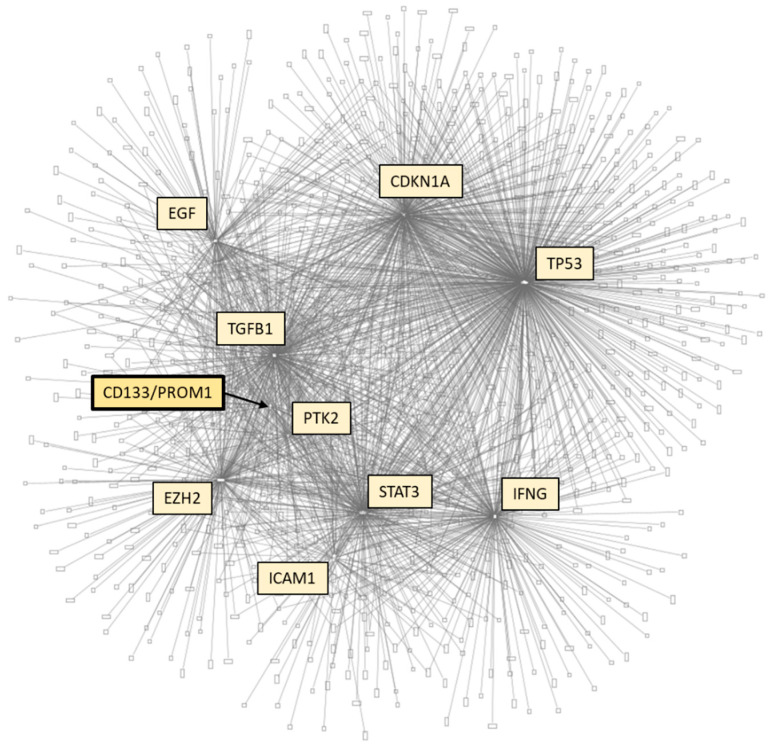
CD133 interaction network in all diseases and biospecimen types based on IPA ((QIAGEN Inc., https://www.qiagenbioinformatics.com/products/ingenuitypathway-analysis) accessed on 24 July 2023) [32].

**Figure A2:**
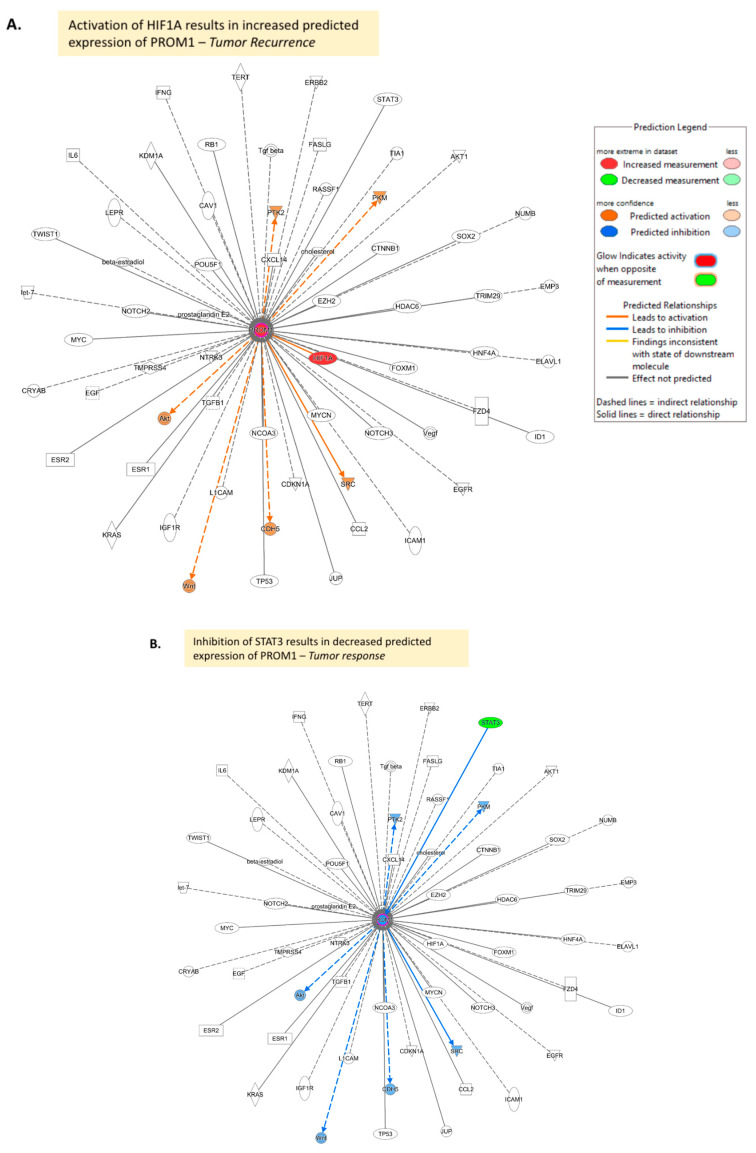
Extended CD133 (PROM1) interaction network limiting interaction to molecules in cancer based on IPA molecule activity prediction when (**A**) HIF1A is activated causing downstream activation and (**B**) STAT3 is inhibited causing downstream inhibition ((QIAGEN Inc., https://www.qiagenbioinformatics.com/products/ingenuitypathway-analysis) accessed on 24 July 2023) [32].

Within these various areas of active research, several pathways have been explored in attempts to expose the relationship of CD133 to GBM cell lines (stem cell and non-stem cell based), tumor tissue, and peripheral blood. A brief summary of the identified molecules and their coinciding connecting signaling pathways is presented here. CD133 is involved in a multitude of pathways, whether directly or indirectly, and having these relationships centralized is useful as the mechanism of action through which CD133 connects to over 3290 molecules [32] and signaling pathways is constantly evolving (Figure A1). However, the most significant connections align with EGF, CDKN1A, p53, TGFB1, PTK2, EZH2, ICAM1, STAT3, and IFNG.

As shown in Figure 2A,B, the direct and indirect downstream connections of CD133 are identical in various scenarios, revealing the multidirectional nature of these relationships. Specifically, these figures demonstrate that the impact of hypoxic conditions, which is shown through HIF activation on one hand and the decreased expression of STAT3 on the other, drive the balance between response and recurrence with CD133 as a functional unit (Figure 2 and Figure 3).

**Figure 4:**
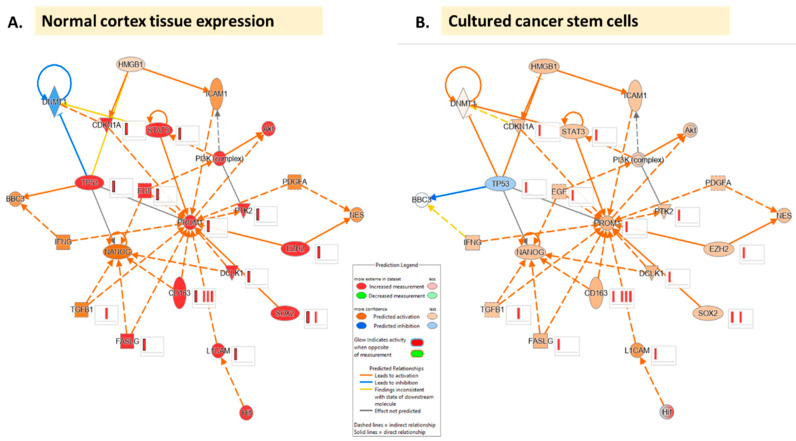
CD133 interaction network based on IPA ((QIAGEN Inc., https://www.qiagenbioinformatics.com/products/ingenuitypathway-analysis) accessed on 24 July 2023) [32]. Network comparison analysis with tissue expression dataset files obtained from IPA, (**A**) normal cerebral cortex and (**B**) cultured cancer stem cells [33].

**Figure A3:**
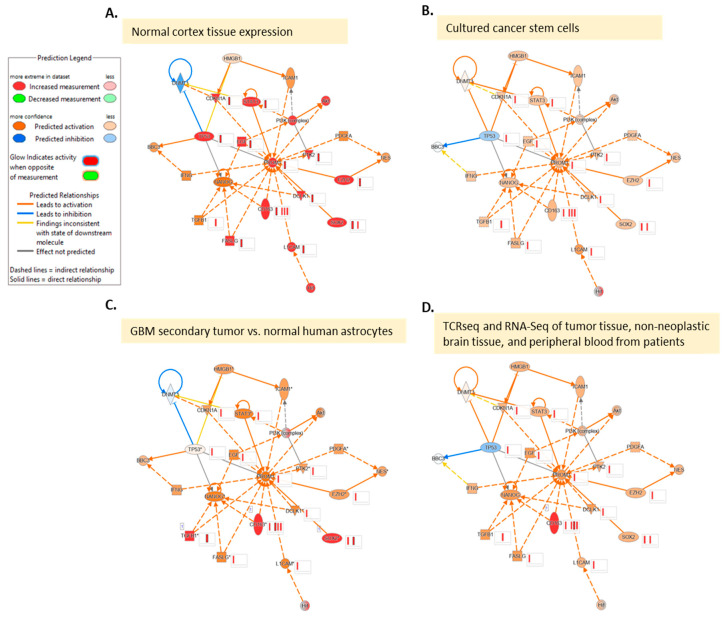
CD133 interaction network based on IPA ((QIAGEN Inc., https://www.qiagenbioinformatics.com/products/ingenuitypathway-analysis) accessed on 24 July 2023) [32]. Network comparison analysis with tissue expression dataset files based on IPA, (**A**) normal cerebral cortex and (**B**) cultured cancer stem cells [33]. (**C**) tumor sample analysis GBM secondary tumor vs. BS149 cell line (normal human astrocytes) [34]. (**D**) TCRseq and RNA-Seq of tumor tissue, non-neoplastic brain tissue, and peripheral blood from patients [35].

Employing the immediate pathway connections of CD133 discussed in previous sections of this review in IPA [32], network maps were generated and overlays of data sets were superimposed to showcase the potential effects on CD133 in a variety of glioma settings for which data were available. This was done with normal cortex tissue expression values based on an IPA dataset (Figure 4A) and using a comparison of GBM stem cells and non-stem tumor cells in culture (Figure 4B) [33]. Two other scenarios were also used which included GBM secondary tumor vs. BS149 cell line [34] and GBM vs. normal control using TCRseq and RNA-Seq of tumor tissue, non-neoplastic brain tissue, and peripheral blood from patients [35] (Figure A3). While there were noticeable similarities in the effects of upstream molecules on CD133, notable differences included the differential effect of p53 on DNA methyltransferase 1 (DNMT1) which, as described in earlier sections, has implications for maintaining cells in a slow cycling state. This state was also cited as having implications for both progression as well as response to TMZ in glioma. Close inspection of the CD133 interaction networks in IPA, as well as other databases, can allow for the identification of other proxy molecules such as DNTM1 that may define the tumor state vs. normal tissue signal in large-scale omic panels to help untangle the complex mechanistic redundancy observed otherwise.

Additionally, several specific pathways have emerged in the topic areas mentioned above that merit focus for future research (Figure 2 and Figure 3). Beginning with prognosis, the affiliation and interplay between HOX genes and CD133 has been suggested [12]. There is also evidence that the Wnt pathway is implicated in signaling with links to metabolic and cell migration [30](Figure 2). Several other links also exist (Figure 3), however, there is a lack of robust investigation into these pathways as further study is required. Regarding the microenvironment, existing data supports links to HIF signaling driving stemness under stress conditions as well as downstream signaling leading to treatment resistance and tumor recurrence [16,36,37]. Also, adaptive changes under hypoxic conditions are linked to VEGF [17]. Looking at resistance, investigation into the PI3K/Akt pathway has begun [22], along with connections to Sox2 [20], PUMA (BBC3) [18], and mTOR [21]. Finally, recurrence studies have explored STAT3 [26], DMNT1 [28], and the HMGB1 gene [24]. It is also significant to note that CD133 expression has been found to be determined by promoter methylation as opposed to immunohistochemical expression in glioma, and promoter methylation status has been associated with recurrence [25,37,38]. The interplay between molecules appears to be affected significantly by the origin of the tissue and sample and the data comparison carried out (Figure 3). Additional markers connected to CD133 are Nestin [9,29,31], Sox2 [20], and Nanog [39] (Figure A3) as well as a number of other emerging markers [24,40]. Finally, when pathway linkages are explored in IPA (Figure A3), it has been noted that CD163, a prognostic biomarker associated with immune response in GBM, also connects to CD133 which represents a promising additional avenue for further research in conjunction with the mediators for which the effect of CD133 is currently not predicted [41,42]. 

## 7. Conclusions

Although there is still a lack of consensus regarding the exact mechanistic relationships of CD133 to prognosis, the tumor microenvironment, resistance, and recurrence, these fields of research are rapidly progressing. The role of CD133 as a functional unit involved in each of these continues to gain momentum, as shown in this review. A comprehensive understanding of the role of CD133 remains elusive but is crucial as omic data becomes increasingly accessible, since CD133 will be included in panels aimed at measuring and classifying tumor resistance. As a result, being aware of its functions and the meaning of its alteration will become incredibly significant due to its potential ability to track prognosis, as well as its possible role as a treatment target. Clarity into which of these mechanisms is most significant creates immense potential for the utilization of CD133 to develop treatment mechanisms, as its inhibition may reduce the tumorigenic and resistance capabilities of GBM. In conclusion, the arrival of large-scale omic data has prompted the need for increased information regarding the role of CD133. This review aimed to highlight the main areas of focus in the research field surrounding CD133 as well as gaps in defined relationships regarding biologic signaling prediction and prognosis, the tumor microenvironment, resistance, and recurrence. Also, currently grey areas of linkage where more research is needed are highlighted in the figures presented. Further advancement regarding the role and linkage of CD133 could allow for additional prognostic and predictive uses as well as specific pathways that can be exploited in treatment approaches and mechanisms in the future.

## Figures and Tables

**Table 1:** Summary and categorization of the articles included in this analysis.

Authors(s)	Category	Title	Overview of Topic
Wu et al., (2015) [9]	Related to Prognosis	*Do relevant markers of cancer stem cells CD133 and Nestin indicate a poor prognosis in glioma patients? A systematic review and meta-analysis*	Association between high CD133 expression and OS and PFS based on the grade of glioma
Han et al., (2016) [10]	Related to Prognosis	*Clinicopathological and Prognostic Significance of CD133 in Glioma Patients: A Meta-Analysis*	Association between high CD133 expression and OS and PFS
Zhang et al., (2016) [11]	Related to Prognosis	*High CD133 Expression Is Associated with Worse Prognosis in Patients with Glioblastoma*	Association of high CD133 expression to OS and PFS
Li et al., (2017) [12]	Related to Prognosis	*CD133 in brain tumor: the prognostic factor*	Connection to HOX gene stem cell factors (HOXA5, HOXA7, HOXA10, HOXC4, HOXC6)
Chavez- Cortez et al., (2019) [13]	Related to Prognosis	*Production and Evaluation of an Avian IgY Immunotoxin against CD133+ for Treatment of Carcinogenic Stem Cells in Malignant Glioma: IgY Immunotoxin for the Treatment of Glioblastoma*	Creation of CD133 immunotoxin from IgY
Abdoli Shadbad et al., (2021) [14]	Related to Prognosis	*The Prognostic Value of CD133 in Predicting Relapse and Recurrence Pattern of High-Grade Gliomas on MRI: A Meta-Analysis*	Association of high CD133 expression with PFS and recurrence
Brown et al., (2017) [15]	Related to Microenvironment	*Expression of CD133 and CD144 in glioblastoma stem cells correlates with cell proliferation, phenotype stability and intra-tumor heterogeneity*	Connection between CD44 and CD133
Lee et al., (2017) [16]	Related to Microenvironment	*CD133 Regulates IL-1Beta Signaling and Neutrophil Recruitment in Glioblastoma*	Relationship between tumor microenvironment and IL-1β signaling pathway
Musah-Eroje et al., (2019) [17]	Related to Microenvironment	*Adaptive Changes of Glioblastoma Cells Following Exposure to Hypoxic (1% Oxygen) Tumour Microenvironment*	Connection between hypoxia and VEGF signaling
Miao et al., (2015) [18]	Related to Resistance	*P53 upregulated modulator of apoptosis sensitizes drug-resistant U251 glioblastoma stem cells to temozolomide through enhanced apoptosis*	Connection between PUMA, TMZ, and CD133
Perazzoli et al., (2015) [19]	Related to Resistance	*Temozolomide Resistance in Glioblastoma Cell Lines: Implication of MGMT, MMR, P-Glycoprotein, and CD133 Expression*	The effect of CD133 on TMZ resistance
Song et al., (2016) [20]	Related to Resistance	*Sox2, a stemness gene, regulates tumor-initiating and drug-resistant properties in CD133-positive glioblastoma stem cells*	SOX2 connection to CD133(+) GBM cells
Sun et al., (2016) [21]	Related to Resistance	*Resistance of glioma cells to nutrient-deprived microenvironment can be enhanced by CD133-mediated autophagy*	CD133 and cell survival via autophagy
Ahmed et al., (2018) [22]	Related to Resistance	*A HIF-independent, CD133-mediated mechanism of cisplatin resistance in glioblastoma cells*	Hypoxia-induced CD133-mediated cisplatin resistance
Juric et al., (2021) [23]	Related to Resistance	*Transcriptional CDK Inhibitors CYC065 and THZ1 Induce Apoptosis in Glioma Stem Cells Derived from Recurrent GBM*	Cyclin-dependent kinases (CDKs) used to treat recurrent glioma
Zhao et al., (2015) [24]	Related to Recurrence	*Effects of HMGB1 on proliferation and apoptosis of human brain glioma CD133 cells*	Relationship between overexpression of HMGB1 gene and CD133
Sun et al., (2016) [25]	Related to Recurrence	*DNA hypomethylation of CD133 promoter is associated with recurrent glioma*	Promoter hypomethylation and glioma recurrence
Chang et al., (2017) [26]	Related to Recurrence	*Synergistic inhibition of tumor growth by combination treatment with drugs against different subpopulations of glioblastoma cells*	Connection to STAT3 and cell viability based on CD133 expression
Polat et al., (2022) [27]	Related to Recurrence	*Differences in stem cell marker and osteopontin expression in primary and recurrent glioblastoma*	Comparison between CD133 expression in original and recurring tumors
Wei et al., (2022) [28]	Related to Recurrence	*The Interaction between DNMT1 and High-Mannose CD133 Maintains the Slow-Cycling State and Tumorigenic Potential of Glioma Stem Cell*	Relation between CD133 and DNA methyltransferase 1 (DNMT1) and its impact on stem cells
Bien-Moller et al., (2018) [29]	Related to Pathways	*Association of Glioblastoma Multiforme Stem Cell Characteristics, Differentiation, and Microglia Marker Genes with Patient Survival*	Connection to CD44, CD95, CD133, ELF4, Nanog, Nestin, and Sparc and OS
Shevchenko et al., (2019) [30]	Related to Pathways	*Proteins of the Wnt signaling pathway as targets for the regulation of CD133(+) cancer stem cells in glioblastoma*	Differences identified in glycolysis/gluconeogenesis, focal adhesion, tight junction and Wnt signaling pathways
Guo et al., (2020) [31]	Related to Pathways	*FRAT1 Enhances the Proliferation of Tumorigenesis of CD133(+) Nestin(+) Glioma Stem Cells In Vitro and In Vivo*	Connection between FRAT1 and CD133 expression

## Data Availability

Not applicable.

## Abbreviations

ATRA	All trans retinoic acid
CNS	Central Nervous System
CDKs	Cyclin-dependent kinases
DNMT1	DNA methyltransferase I
FRAT1	Frequently rearranged in advanced T cell lymphomas-1
GBM	Glioblastoma Multiforme
IPA	Ingenuity Pathway Analysis
OS	Overall Survival
PFS	Progression-free survival
PRPS1	Phosphoribosylpyrophosphate synthetase 1
TMZ	Temozolomide
